# How well will you FIT? Use of a modified MMI to assess applicants’ compatibility with an emergency medicine residency program

**DOI:** 10.3402/meo.v21.29587

**Published:** 2016-02-02

**Authors:** Alice A. Min, Aaron Leetch, Tomas Nuño, Albert B. Fiorello

**Affiliations:** 1 Department of Emergency Medicine, College of Medicine, The University of Arizona, Tucson, AZ, USA; 2 Department of Emergency Medicine and Pediatrics, College of Medicine, The University of Arizona, Tucson, AZ, USA

**Keywords:** MMI, emergency medicine, residency interview

## Abstract

**Purpose:**

Emergency medicine residency programs have evaluated the use of Multiple Mini Interviews (MMIs) for applicants. The authors developed an MMI-style method called the Fast Interview Track (FIT) to predict an applicant's ‘fit’ within an individual residency program.

**Methods:**

Applicants meet with up to five residents and are asked one question by each. Residents score the applicant using a Likert scale from 1 to 5 on two questions: ‘How well does the applicant think on his/her feet?’ and ‘How well do you think the applicant will fit in here?’. To assess how well these questions predicted a resident's ‘fit’, current residents scored fellow residents on these same questions. These scores were compared with the residents’ interview FIT scores. A postmatch survey of applicants who did not match at this program solicited applicants’ attitudes toward the FIT sessions.

**Results:**

Among the junior class, the correlation between interview and current scores was significant for question 1 (rho=0.5192 [*p=*0.03]) and question 2 (rho=0.5753 [*p=*0.01]). Among seniors, Spearman's rho was statistically significant for question 2, though not statistically significant for question 1. The chi-square measure of high scores (4–5) versus low scores (1–3) found a statistically significant association between interview and current scores for interns and juniors. Of the 29 responses to the postmatch survey, 16 (55%) felt FIT sessions provided a good sense of the program's personality and only 6 (21%) disagreed. Nine (31%) felt FIT sessions positively impacted our program's ranking and 11 (38%) were ‘Neutral’. Only two (7%) reported that FIT sessions negatively impacted their ranking of our program.

**Conclusions:**

FIT provided program leadership with a sense of an applicant's ‘fit’ within this program. Interview day scores correlated with scores received during residency. Most applicants report a positive experience with FIT sessions. FIT provides a useful tool to recruit applicants who fit with the residency program.

Research on the use of the Multiple Mini Interview (MMI) for residency interviews has grown significantly over the last year. In September 2014, Phillips and Garmel drew attention to the use of the MMI in emergency medicine (EM) interviews and the applicant's perspective of the process (1). A social media-based discussion about this article was conducted on Academic Life in Emergency Medicine (ALiEM) (2). From this discussion emerged the need for a program's interview day to accomplish three goals: 1) predict which candidates will be successful in emergency medicine; 2) portray the program's personality and strengths accurately; and 3) optimally match the applicant and program (3).

The MMI was developed as 10 stations similar to objective structured clinical examinations (OSCE) with the intention of measuring non-cognitive skills that may not be adequately assessed during a personal interview (4). It has been used extensively for medical school admission interviews. There is a growing body of research on the use of MMIs for residency interviews. Many studies have been conducted in Canada and over a range of medical specialties including otolaryngology, OB/GYN, pediatrics, internal medicine, and family medicine (5–8).

Within the specialty of EM, the MMI has been a growing area of interest. Studies recently published on EM applicants’ perception of the MMI have not been favorable. Hopson et al. conducted an eight-station EM-focused MMI focused with 71 EM interns from three different residency programs (9). They found that the MMI scores correlated with EM clerkship grades but not with match desirability ranking. Also, more participants reported that the MMI would have a negative rather than positive effect on their decision to rank the program highly.

Another study by Soares et al. compared the MMI with traditional interviews for applicants to one EM residency program. On an anonymous postinterview questionnaire, 89% reported that they found the traditional interview more enjoyable than the MMI process, though only 14% of applicants in this study reported that the MMI would have a negative impact on their ranking of the program (10).

The interview day has been shown to be the most influential factor on an applicant's residency selection that is within the residency program's control (11, 12). Because program leadership cannot change the residency's geographic location or its proximity to an applicant's social support system, the interview day is the program's best opportunity to provide an overall attractive experience through showcasing its residents and academic reputation (11). Five aspects of an interview day that seem to be most valuable to applicants include the overall ‘feel’ or ‘personality’ of the program, informal off-campus gatherings with residents, an interview with the program director, meeting with the program coordinator, and interviewing with residents (12).

In order to optimize the interview day experience at our program, we developed an interview process called the Fast Interview Track (FIT). It is a less structured style of the MMI and has come to be nicknamed ‘resident speed dating’. The goal of this experience is twofold. First, it provides more time to each applicant to talk with our residents one-on-one to display the personality of our program; exposing candidates to a larger number of our current residents. Second, it provides more ‘data’ for the residency leadership when formulating our rank list and gives current residents input into our overall rank decision. We hypothesize that the FIT scores predict how well the applicant will fit in with our program. We compared the FIT scores our residents received when they were actual residents in the program with their scores on their residency interview day to examine if we are improving our ability to select applicants that fit in well.

## Methods

We have used the FIT format in our standard interview day since the 2011 interview season. The applicants meet with up to five of our current residents and are asked one different question at each station. These questions are selected from a bank of questions approved by the residency leadership. We developed a mix of questions, some highlighting individual candidate's personal strengths, some making the candidates to think something through to highlight their thought process, and others challenging candidates to discuss a personally perceived negative characteristic trait. On interview day, program leaders choose the five questions to be used and distribute them to the residents, mixing the question types so that all resident–candidate interactions are not biased toward one type of question (Table 1).

**Table 1:** Sample FIT questions

How would your friends describe you? What are you most proud of? What is one thing about yourself that you would like me to know? What do people most criticize about you? At certain times, everyone has to work with a person where there is a serious clash of personalities. How do you handle a situation where there is a mutual dislike? Give an example of a time you felt you went above and beyond the call of duty. What is your greatest failure and what did you learn from it? What irritates you about other people? How do you deal with it? If I were your supervisor and asked you to do something that you disagreed with, what would you do? Tell me about a mistake you made and how you handled it. We are often very busy and have too many tasks to complete in a given amount of time. How do you handle this? Have you ever gotten bad feedback? How did you handle it? Have you ever been part of a team where a member was not pulling their weight? How did you handle it?

An applicant meets with each resident individually for 5 min in a ‘speed-dating’ format. The resident then scores the applicant on two questions:

1. How well does the applicant think on his or her feet?
2. How well do you think the applicant will fit in here?

Scores are based on a Likert scale anchored by 1=poor and 5=best. These scores are then incorporated into the applicant's overall interview score, which the program leadership then uses to determine our rank list. Per our standard interview scoring process, we average the scores the applicant receives on each of the two questions; then take the sum with a maximum score of 10. The first year we instituted the FIT, corresponding to the current senior residents, we used the mean of the two scores; therefore, the maximum score was five for the first year of its use. We performed this study at a 3-year academic emergency medicine program with 45 categorical EM residents and 8 combined EM/pediatrics residents. This study was reviewed by the local institutional review board and deemed exempt. During the time the FIT sessions were utilized, 44 of the 45 categorical residents and 8 of the 9 combined EM/Pediatric residents participated in the FIT sessions during their interview day. We surveyed all current residents via SurveyMonkey and asked each resident to score their fellow residents in the two classes other than their own on the same two questions used in the FIT session. This survey was conducted in January, after the interns had been in the program for 6 months. The scores from these surveys were compared with the FIT scores that each current resident received on their actual interview day.

The primary outcomes for this study were Spearman rank correlation coefficients between interview scores and follow-up scores to measure the strength of association between the two ranked variables. Given the non-linear relationship between variables, the Spearman rank correlation was chosen over the Pearson correlation coefficient. In addition, mean interview and current scores for both questions were assessed by paired *t* tests, as previous studies have shown that for five-point Likert items, the *t* test and its non-parametric counterpart the Mann–Whitney–Wilcoxon generally have similar power. Lastly, chi-square tests of association were conducted for the combined questions during interview and follow-up assessments in order to assess an overall association between interview and current scores. All statistical tests were two sided at a significance level of 0.05. No adjustments were made for multiple comparisons. All analyses were performed using Stata version 14.0 (StataCorp, College Station, TX).

We also conducted a postmatch survey of our top ranked applicants that did not match at our program, which is our standard practice each year. Among other questions, we asked the following questions specifically pertaining to the FIT sessions:

1. Did the FIT sessions give you a good sense of the personality and feel of our program and residents? (Yes or No)
2. How did the FIT sessions impact your ranking of our program? (Negatively, Neutral, or Positively)

## Results

Of the 52 residents surveyed, 44 were categorical EM residents and 8 were EM/pediatrics residents. There was an even split between males and females that participated with 26 female and 26 male (Table 2).

**Table 2:** Baseline Demographics

Variable	Interns (*n=*18)	Juniors (*n=*18)	Seniors (*n=*16)
Men (%)	56%	50%	44%
Women (%)	44%	50%	56%
Question 1: How well does the applicant think on his or her feet?*			
Score on interview day	4.28±0.52	4.12±0.51	4.23±0.45
Score as current resident	4.15±0.32	3.88±0.51	4.18±0.58
	*p=*0.34	*p=*0.05	*p=*0.76
Question 2: How well do you think the applicant will fit in here?*			
Score on interview day	4.18±0.63	3.92±0.72	4.23±0.45
Score as current resident	4.33±0.45	4.12±0.55	4.31±0.60
	*p=*0.27	*p=*0.15	*p=*0.58

* Values are means±standard deviation.

Among all three groups, the mean score for question 1 was lower on the current assessment compared to the interview day assessment. This result was statistically significant among juniors but not among interns and seniors. The difference was largest among juniors, with a mean interview score of 4.12 (SD=0.51) compared with a mean current score of 3.88 (SD=0.51). However, the mean score for question 2 was higher during the current assessment compared to interview day assessment. There was no statistical significance among any groups for question2. The largest difference was again among the juniors. Among seniors, there was a data limitation for question 2.

Correlations between baseline and follow-up scores are shown in Table 3. A significant correlation was found among junior class interview day and current scores for both questions. For question 1, Spearman's rho was equal to 0.5192 (*p=*0.03), and for question 2, rho was equal to 0.5753 (*p=*0.01). Thus, among juniors, a strong and significant positive correlation was found. We rejected the null hypothesis of no association between interview and current scores and concluded that interview and current scores have a positive relationship (as one increases, the other also increases). Among seniors, Spearman's rho was also fairly strong and statistically significant for question 2, though not statistically significant for question 1.

**Table 3:** Spearman correlation coefficients of interview scores with current scores

Interns	Juniors	Seniors
Q1: *ρ*=0.1137, *p=*0.65	Q1: *ρ*=0.5192, *p=*0.03	Q1: *ρ*=0.4077, *p=*0.12
Q2: *ρ*=0.2122, *p=*0.40	Q2: *ρ*=0.5753, *p=*0.01	Q2: *ρ*=0.4974, *p=*0.05

Among interns, Spearman's rho was weak, indicating that there was no tendency for the current score to be either higher or lower when the interview day score was higher (Table 3).

Table 4 presents another measure of association between the overall association among interview day and current scores. When categorized as high (scores of 4 and 5) and low (scores of 1, 2, and 3) interview and current scores, the chi-square measure of association was used to assess whether the relationship between interview and current scores was due to chance or if the relationship was systematic. We combined both questions for this analysis to assess the overall association. For interns and juniors, we found a statistically significant relationship (Interns: *χ*^2^=9.05, *p=*0.003; Juniors: *χ*^2^=5.60, *p=*0.02) between interview and current scores, indicating our findings are unlikely to occur if there was no association between interview and current scores. Among juniors, this further strengthens the findings from the Spearman correlation analyses. Among interns, the results are mixed but could indicate that if the sample size were larger, results from the Spearman correlation analyses could have been stronger. For seniors, the chi-square results did not reach significance, though this result did have data limitations described in the discussion section. For interns, our calculation of odds ratios showed that for those with high interview scores, the odds of a current high score were 11.5 times larger than the odds for those with a low interview score (OR=11.5; 95% CI, 2.2–62.0) These results were similar for juniors (OR=5.5; 95% CI, 1.3–22.7) but not for seniors (OR=3.7; 95% CI, 0.5–26.0) (Table 4).

**Table 4:** Association between low and high interview and current scores

		Interns (current)	
		
		Low	High

Interns (interview)	Low	6	4
	High	3	23
*χ*^2^=9.05, *p=*0.003; (OR=11.5; 95% CI, 2.2–62.0)			

		Juniors (current)	
		
		Low	High

Juniors (interview)	Low	11	4
	High	7	14
*χ*^2^=5.60, *p=*0.02; (OR=5.5; 95% CI, 1.3–22.7)			

		Seniors (current)	
		
		Low	High

Seniors (interview)	Low	2	2
	High	6	22
*χ*^2^=1.52, *p=*0.22; (OR=3.7; 95% CI, 0.5–26.0)			

### Survey results

We sent postmatch surveys to 49 highly ranked applicants that did not match into our program. We asked two questions about the FIT interview on this survey. We received a total of 29 responses (59%).

Regarding the question ‘Did the FIT sessions give you a good sense of the personality and feel of our program and residents?’, 16 (55%) responded ‘Yes’ and 6 (21%) responded ‘No’. Seven (24%) respondents skipped this question. For the question ‘How did the FIT sessions impact your ranking of our program?’, 9 (31%) responded ‘Positively’, 11 (38%) responded ‘Neutral’, and 2 (7%) reported that the FIT sessions negatively impacted their ranking of our program. Seven respondents again skipped this question (Table 5).

**Table 5:** Postmatch survey questions

		Yes	No	Skipped
Did the FIT sessions give you a good sense of the personality and feel of our program and residents?		16 (55%)	6 (21%)	7
		Positively	NeutralNegatively	Skipped
How did the FIT sessions impact your ranking of our program?		9 (31%)	11 (38%)2 (7%)	7 (24%)
		Positively	Neutral	Negatively
Response to ‘How did the FIT sessions impact your ranking of our program?’		I liked the 5 minute speed dating component with residents. Although it wasn't much time to have a meaningful conversation, I think these 5 minutes are important in EM and show a program how the applicant reacts in short time periods.	FIT sessions are a little jarring since they were a combo of ‘get to know you’ and situational questions that didn't seem to match up, but certainly non-confrontational and quite pleasant.	I would consider removing the resident rotating interviews.
		Best interview day. I attended in terms of communicating values of the program and introducing program residents and faculty.	The ‘speed dating’ with the residents was a little odd and was mostly generic interview questions, which I don't feel helped me understand your program or help you understand me very well.	The only thing I didn't enjoy was the rapid resident interview sessions after the regular interviews. It was a little tiring and it didn't seem that enjoyable to several of the residents either. Also the interactions were too short to gain any sense of the people/program and they had standardized questions, which made it feel more like an interview than anything else.
		Really liked being able to interact and interview with residents as well as faculty.	… I was a little turned off by the ‘speed dating’ … That said, this had no bearing on my rank list.	
		The FIT sessions helped me feel like the residents got an idea of what I would be like to work with.	Really enjoyed dinner with residents, mini interviews, …	
		My favorite part was the speed dating with the residents.		
		I also enjoyed the mini interviews with the residents.		

We ask an open-ended question in our survey that states ‘How did our interview day compare to the top 5 programs on your rank list? How would you suggest improving it?’. Comments related to the FIT sessions are noted in Table 5.

## Discussion

Optimizing the interview day experience, and specifically the use of the MMI, is a topic of interest in emergency medicine. This is evidenced by the growing number of studies on the use of MMI for resident selection within our specialty and the social media discussion held on ALiEM (2). Opinions shared on this platform were varied, but most seemed to feel that the MMI was a forced, overly structured interaction that leaves the applicant feeling stressed and without a true sense of the program (3). While the MMI may provide some standardization to the interview scores, it may risk depersonalizing the program.

We have attempted to create an interview experience that benefits all stakeholders. Joshi et al. identify three goals of the interview day: 1) predict which candidates will be successful in emergency medicine; 2) portray the program's personality and strengths accurately; and 3) optimally match the applicant and program (3). We feel our FIT sessions address all three of these goals. We assess the applicant's ability to think on their feet. The sessions allow the applicants to interact with multiple residents and get a broader exposure to the personality of the residents and program. The program leadership gets scores and comments from more individuals to help assess how desirable they are to our program and if our program would be a good match for them.

Our study demonstrates a good correlation between the scores our residents receive in the FIT sessions on their actual interview day and the scores they receive from their fellow residents when they are actually members of our program. For the junior class, a strong correlation and statistically significant association was found between the interview day and current FIT scores. For the intern class, the correlation analyses were not statistically significant, and the magnitude of the correlation was low. However, the chi-square test of association was statistically significant, indicating a larger sample size for the correlation analysis could have more power. Additionally, the interns were only in our program for 6 months, some with little actual time working in the emergency department, when the current residents were asked to evaluate how well they fit in. The association of the senior class scores did not reach significance, though the magnitude of the association was fairly strong. The analysis of the scores for the senior class was limited by the fact that their interview day scores were an average of the scores on the two FIT assessment questions. The scores for each individual question were not available.

The strongest results were among juniors. For question 1 regarding thinking on their feet, the statistically significant slight decrease could be due to the current assessments taking place after seeing juniors work professionally. An interesting trend, though not statistically significant, was that the mean score for question 2 was higher during the current assessment compared to the interview day assessment. This question asks how well an individual fits in with our program.

A possible explanation is that ‘fit’ could be affected by professional relationships that have developed between the interview and current score dates.

The majority of the postmatch survey responses related to the FIT sessions were positive. Someone even commented regarding their interview day with our program that their ‘favorite part was the speed dating with the residents’. Only two respondents reported that the FIT sessions had a negative impact on their ranking of our program, while nine felt it had a positive impact. Some comments from those that reported a neutral effect on their ranking had a negative tone; however, the majority of the comments were positive overall.

Based on these results, we will continue to incorporate the FIT sessions into our interview day. Although the structured MMI used in other studies may have left applicants without a sense of personality and ‘feel’ of the program, our unique FIT sessions provide applicants with a more comprehensive view of our program and its personality. This format helps us showcase our residents and also allows for residency leadership to gauge the sentiments of our current residents toward the applicants. Our goal is to create a good fit between applicant and program, ultimately leading to a happy group of residents to carry on the FIT sessions and help recruit future residents who will fit in well with us.

## Limitations

Our study findings are limited by a small sample size and single residency program. We did not compare our FIT scores with other variables of the application including United States Medical Licensing Examination (USMLE) score or clerkship grades. We also did not compare the FIT scores with performance measures during residency training such as In-Training exam scores, evaluations on clinical rotations, or milestones assessing professionalism. This is something that we plan to investigate in the future.

The data analysis of the senior class was limited by the fact that we did not have the individual FIT scores for each of the two questions. We had only the average of the two as this is what was recorded for the assessment of applicants that year. The interns had only been a part of our program for 6 months when current residents evaluated how well they fit in. This may not have been enough time to adequately assess this.

The postmatch survey responses are from a small sample of applicants. We sent out 49 surveys, and 29 were completed and returned. Only 22 people responded to the questions specifically addressing the FIT sessions. This number represents about 20% of the total applicants we interviewed.

## Conclusions

The FIT sessions provide the program leadership and residents with a sense of how an applicant will fit in with our residency program. Most applicants have a positive experience in the FIT sessions. Any device or method that helps align the personalities and goals of residency programs and applicants is of benefit to all stakeholders.

One question that may be raised with this process is, Does utilizing peer evaluation to ensure a good ‘fit’ in a program discourage diversity? We have not found this to be the case. Our approach to applicant selection and candidate ranking is holistic, without rigid selection or rejection criteria. The FIT interview score is a portion of the overall evaluation of an applicant, currently constituting only 10% of the overall score we use to arrive at our initial ranking. We consider the applicant's entire portfolio when determining where a candidate ends up on our final match list. We believe that the 10% contribution of FIT to the candidate's score adds value in our overall assessment and a different perspective of the candidate that was not present with application review and traditional interviews alone. In addition, the process allows the candidates to interact with more of our current residents and these residents to be engaged, contributing to our selection process. With proper training of residents on the interview process and utilizing a holistic approach, we feel the FIT interviews can enhance camaraderie and cohesiveness without detrimentally affecting the diversity of a program.

The FIT session is a method that has proven beneficial for us and may be beneficial to other programs as well.
